# Osteopenia and the physical function in Japanese patients with schizophrenia

**DOI:** 10.1007/s11657-017-0391-7

**Published:** 2017-10-27

**Authors:** Satoru Uchida, Tsuyoshi Ichinose, Yoichi Iizuka, Koichi Okamura, Hitoshi Shitara, Manabu Yamazaki, Kenji Takagishi, Haku Iizuka

**Affiliations:** 1 Department of Orthopaedic Surgery, Saint-Pierre hospital, Takasaki, Gunma Japan; 2 Department of Orthopaedic Surgery, Gunma University Graduate School of Medicine, 3-39-15 Showamachi, Maebashi, Gunma 371-8511 Japan; 3 Department of Psychiatry, Saint-Pierre hospital, Takasaki, Gunma Japan

**Keywords:** Osteopenia, Schizophrenia, Physical function

## Abstract

***Summary*:**

We evaluated the state of osteopenia and the physical function in 121 schizophrenic patients. These factors were worse in the inpatient group than in the outpatient group. The age, sex, body mass index (BMI), and physical function were correlated to the state of osteopenia. Physicians should consider the risk of osteopenia in elderly female psychiatric patients with low BMI.

**Purpose:**

Information about the actual state of osteopenia in patients with schizophrenia is limited. In the present study, we evaluated the factors related to osteopenia and patient’s physical function and compared these factors between inpatients and outpatients.

**Methods:**

A total of 121 schizophrenic patients were included in the present study. We divided the patients into two groups according to the therapeutic form. We collected data on their age, sex, body mass index (BMI), bone mineral density (BMD) in the lumbar spine and proximal femur, serum bone metabolic markers, risk of fracture, and physical function.

**Results:**

The number of fractured vertebrae, risk of fracture, serum concentration of tartrate-resistant acid phosphatase 5b (TRACP-5b), and score of locomo25 were significantly higher and the BMI and BMD in the lumbar spine and proximal femur significantly lower in the inpatient group than in the outpatient group. A multiple regression analysis showed that the age, sex, BMI, the number of fractured vertebrae, and score of locomo 25 were correlated with the BMD in the lumbar spine and proximal femur. Neither the therapeutic form nor any bone metabolic markers were correlated with the BMD. The inpatient group had a lower average BMI, BMD, and physical function than the outpatient group. However, a multiple regression analysis showed that the therapeutic form was not correlated with the BMD.

**Conclusion:**

These findings suggest that physicians should consider elderly female schizophrenic patients with a low BMI to be at risk of developing osteopenia.

## Introduction

Osteopenia is a common musculoskeletal disorder in the elderly considered to be caused by a combination of physical, metabolic, and endocrine factors [1, 2]. A decreased physical activity may reduce the mechanical loading to bone or skeletal muscle [3] and result in a low bone mineral density (BMD) [2, 4]. A decreased or abnormal metabolism of calcium or collagen may affect the bone quality [1]. Changes in the synthesis of a sex steroid hormone may also affect the bone metabolism [1]. These changes are caused by aging in many cases. However, osteopenia can occur even in young people if the above conditions are met by special circumstances or induced by disease.

Many patients with psychiatric disorder, such as schizophrenia or depression, have to be hospitalized for a long time in Japan. Several studies have reported a decline in the physical function of elderly patients due to hospitalization [5, 6]. Therefore, we expected that long-term hospitalized patient with psychiatric disorder would also show a decline in their physical function. A low physical function may cause disuse atrophy in various organs, skeletal muscle, and bone. When such disuse atrophy occurs, patients cannot be easily discharged from the hospital, even when the psychiatric disorder has improved. However, whether or not the physical function of patients with psychiatric disorders is different between inpatients and outpatients remains unclear. In addition, some drugs for psychiatric disease, especially those for schizophrenia, affect hormone production. In particular, drug-induced prolactin (PRL) production is thought to reduce the sex steroid hormone production via a negative feedback cascade [7], resulting in osteopenia. Therefore, clarifying the actual state of osteopenia in patients with psychiatric disorders is important for creating a comprehensive treatment plan.

The aims of this study were to evaluate the state of osteopenia and its related factors in patient with schizophrenia and to compare these factors between inpatients and outpatients. We hypothesized that inpatients have a higher risk for osteopenia and a low physical function than outpatients and that the therapeutic form affects the state of osteopenia in schizophrenic patients.

## Materials and methods

### Subjects and study design

This study was approved by the ethics committee of Gunma University, and written consent was obtained from all patients and their family or guardian.

**Fig. 1:**
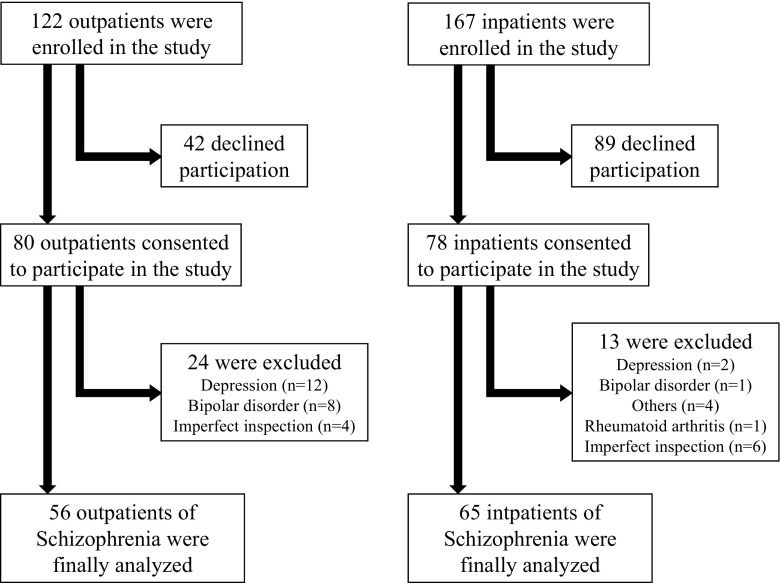
The cascade of selection of participants

The subjects were patients treated for schizophrenia at a regional psychiatric hospital. The diagnosis of the patients was determined by a psychiatric specialist according to the DSM-IV. The cascade of selection of participants is shown in Fig. 1. Briefly, we recruited 122 outpatients and 167 inpatients for this study. We excluded 42 outpatients and 89 inpatients because they declined to participate. We further excluded 24 outpatients and 13 inpatients because they had other psychiatric disorders, such as depression, and comorbidities like rheumatoid arthritis, or because they could not complete all of the examination. We ultimately included 59 outpatients and 65 inpatients and compared the examination results described below between the two groups.

### Basic information about physical and medication

We measured the height and body weight of all participants and recorded the type of medication (typical or atypical) and daily dose. The body mass index (BMI) and chlorpromazine (CP) equivalent were calculated from this information.

### BMD examinations

All participants underwent a BMD examination in their lumbar spine and proximal femur with a dual X-ray absorption measurement device (Prodigy; GE Healthcare, Solingen, Germany). If the patient had a history of surgical treatment for unilateral proximal femoral fracture, the BMD examination was measured on the opposite side; patients with a history of implantation for the lumbar spine or surgical treatment for bilateral proximal femoral fracture were excluded from the BMD examination. The BMD (g/cm^3^), young adult mean (%), and T score were calculated at each examination site.

### X-ray examinations

All participants underwent an X-ray examination of their thoracic and lumbar spine to evaluate the history of vertebral fracture. Fracture was defined as a deformity of grade I or more according to the classification by Genant [8]. In the present study, one or more fractured thoracic or lumbar vertebrae constituted a history of fracture.

### Diagnosis of osteoporosis

We diagnosed participants with osteoporosis based on the following criteria [9]: a T score in the lumbar spine or proximal femur of less than − 2.5 and the presence of vertebral fracture at the time of the X-ray examination. If a participant met both criteria, we diagnosed them with severe osteoporosis.

### FRAX

FRAX is a tool developed to calculate the risk of proximal femur fracture or major osteoporotic fracture (spine, forearm, hip joint, and shoulder) within the next 10 years. This tool consists of 12 items [10, 11], and the information needed to perform the calculation was obtained from the participants and their examination results.

### Blood sampling

All participants underwent blood sampling for inspection of the following: serum concentration of type I procollagen N-terminal propeptide (PINP), tartrate-resistant acid phosphatase 5b (TRACP-5b), hemoglobin (Hb), albumin (Alb), calcium (Ca), and prolactin (PRL). We were unable to obtain data on PRL from all participants because some declined to give their consent.

### The physical function

We evaluated the patients’ physical function using the locomo 25 [12, 13]. The locomo 25 is a self-reported, relatively comprehensive measure that consists of 25 items, including 4 questions regarding pain during the last month, 16 regarding activities of daily living during the last month, 3 regarding social functions, and 2 regarding mental health status during the last month. These 25 items are graded on a 5-point scale from no impairment (0 points) to severe impairment (4 points) and then arithmetically summed to produce a total score (minimum 0, maximum 100). Thus, a higher score is associated with a worse physical function, and the cut-off score for identifying locomotive syndrome was set at 16 [13]. In the present study, considering the impaired self-assessment ability of the patients, well-trained medical staff also delivered the locomo 25, and we evaluated the differences in the results between the self-answered version and the staff-administered version.

### Statistical analyses

The SPSS software program (version 24.0; IBM, Chicago, IL, USA) was used for the statistical analyses. Data were subjected to the independent *t* test, Chi-squared test. In addition, univariate regression analysis was used to test the association of one variable with the BMD in the lumbar spine and proximal femur without considering other variables or confounders. The independent variables were as follows: age, sex (0 = male, 1 = female), BMI, hospitalization (treatment) duration, CP equivalent, usage of typical antipsychotic drug (0 = no use, 1 = use), the number of fractured vertebrae in the lumbar spine, the score of locomo 25, and the serum concentration of PINP, TRACP-5b, Hb, Alb, Ca, and PRL. We also performed a multiple regression analysis with confounding factors, using the stepwise selection approach.

## Results

### Difference in factors between inpatient and outpatient groups

A total of 121 schizophrenic patients were enrolled in the present study. Of these, 65 were inpatients, and 56 were outpatients. As shown in Table 1, the mean treatment duration in the inpatient group was 236.5 months, while that in the outpatient group was 138.0 months. The distribution of sexes was not markedly different between the two groups. However, the mean age was significantly higher in the inpatient group than in the outpatient group, and the mean BMI was significantly lower in the inpatient group than in the outpatient group.

**Table 1:** Comparison of parameters between inpatient and outpatient groups

	Inpatient (*n* = 65)	Outpatient (*n* = 56)	*p* value
Hospitalization/treatment duration (month)	236.5 ± 28.4	138.0 ± 26.4	0.013
Age	63.2 ± 1.5	58.5 ± 1.7	0.046
Sex (male/female)	23/42	29/27	0.051
Body mass index (BMI)	20.2 ± 0.4	25.1 ± 0.5	< 0.01
CP equivalent (mg/day)	433.2 ± 45.4	357.1 ± 45.1	0.241
Antipsychotic drug (atypical/typical)	23/40	30/24	0.030
%	63.5	44.4	
BMD in lumbar spine (g/cm^2^)	0.91 ± 0.02	1.05 ± 0.02	< 0.01
YAM in lumbar spine (L1-L4) (%)	81.1 ± 2.2	91.9 ± 2.0	< 0.01
T score in lumbar spine (L1-L4)	− 1.77 ± 0.20	− 0.75 ± 0.19	< 0.01
BMD in proximal femur (g/cm^2^)	0.71 ± 0.02	0.87 ± 0.02	< 0.01
YAM in proximal femur (%)	76.0 ± 2.1	92.9 ± 2.1	< 0.01
T score in proximal femur	− 1.82 ± 0.16	− 0.53 ± 0.16	< 0.01
Count of vertebral fracture (Th)	0.9 ± 0.2	0.1 ± 0.1	< 0.01
Count of vertebral fracture (L)	0.7 ± 0.1	0.2 ± 0.1	< 0.01
Count of vertebral fracture (total)	1.6 ± 0.3	0.4 ± 0.1	< 0.01
Diagnosis of osteoporosis (N/Y)	37/28	50/6	< 0.01
Diagnosis of osteoporosis (vertebral fracture) (N/Y)	21/44	42/14	< 0.01
Severe osteoporosis (N/Y)	43/22	54/2	< 0.01
Medical staff-evaluated score of locomo25	25.5 ± 2.5	14.4 ± 2.0	< 0.01
Positive rate of locomotive syndrome medical staff evaluated (%)	60.9	33.9	< 0.01
Self-evaluated score of locomo25	20.1 ± 1.9	14.4 ± 2.0	0.041
Positive rate of locomotive syndrome self evaluated (%)	52.4	33.9	0.033
Discrepancy between medical staff evaluation and self-evaluation	5.44 ± 1.63	0.04 ± 0.03	< 0.01
FRAX component (N/Y)			
Past history of fracture	47/17	37/19	0.248
Proximal femoral fracture of parents	61/3	52/3	0.586
Current smoking	64/0	45/11	< 0.01
Glucocorticoid	62/2	55/1	0.550
Rheumatoid arthritis	62/2	55/1	0.550
Secondary osteoporosis	62/2	54/2	0.639
Alcohol intake	64/0	54/2	0.216
Risk ratio of major osteoporotic (%)	12.9 ± 1.6	7.6 ± 0.9	< 0.01
Risk ratio of hip fracture (%)	4.7 ± 1.2	1.5 ± 0.4	0.011
Hb (g/dl)	12.5 ± 0.2	13.4 ± 0.2	< 0.01
ALB (mg/dl)	4.1 ± 0.0	4.4 ± 0.0	< 0.01
Ca (mg/dl)	9.2 ± 0.1	9.5 ± 0.1	< 0.01
TRACP-5b (mU/dl)	491.1 ± 33.7	335.8 ± 21.5	< 0.01
Total P1NP (μg/l)	60.0 ± 3.0	54.5 ± 3.2	0.213
PRL (ng/ml)	50.1 ± 4.3 (*n* = 52)	38.9 ± 16.1 (*n* = 11)	0.512

The CP equivalent was not markedly different between the two groups; however, the rate of administration of typical antipsychotic drugs was significantly higher in the inpatient group than in the outpatient group. The BMD, YAM, and T score in the lumbar spine and proximal femur were significantly lower in the inpatient group than in the outpatient group. The number of fractured vertebrae was significantly higher in the inpatient group than in the outpatient group. The ratio of the diagnosis of osteopenia was significantly higher in the inpatient group than in the outpatient group. The risk ratio of proximal femur fracture and major osteoporotic fracture was both significantly higher in the inpatient group than in the outpatient group. The serum concentrations of PINP and PRL were not significantly different between the two groups; however, the serum concentrations of Hb, Alb, and Ca were significantly lower in the inpatient group than in the outpatient group, while the serum concentration of TRACP-5b was significantly higher in the inpatient group than in the outpatient group.

Discrepancies in the results of the locomo 25 between the self-evaluated and staff-administered versions were more evident in the inpatient group than in the outpatient group. The inpatient group tended to overestimate their physical function, and the greatest difference between the self-answered score and staff-evaluated score was 58 points in the inpatient group and 1 point in the outpatient group. The self-answered score of the locomo 25 and staff-evaluated score were significantly higher in the inpatient group than in the outpatient group. Furthermore, the positive rate of locomotive syndrome was significantly higher in the inpatient group than in the outpatient group (Table 1). Regarding the inter-rater reliability of the evaluation of locomo 25, the kappa coefficient was 0.96 in the outpatient group and 0.59 in the inpatient group. We therefore decided not to use the self-evaluated results of locomo 25, considering the low reliability in the inpatient group, and instead used the medical staff-evaluated results for subsequent analyses.

### Univariate and multiple regression analyses

In the univariate regression analysis, the age, sex, BMI, treatment form, staff-evaluated score of the locomo 25, positive rate of locomotive syndrome in the medical staff evaluation, number of fractured vertebrae in the lumbar spine, and serum concentrations of Hb, Alb, PINP, and TRACP-5b were significantly correlated with the BMD in the lumbar spine. In addition, these factors, along with the serum concentration of Ca, were significantly correlated with the BMD in the proximal femur (Table 2).

**Table 2:** The results of univariate analysis

BMD lumbar spine (g/cm^2^)		
Factor	β	*p* value
Age	− 0.441	< 0.01
Female	− 0.424	< 0.01
BMI	0.529	< 0.01
Inpatient	− 0.327	< 0.01
The number of fractured vertebrae (L-spine)	− 0.441	< 0.01
The score of locomo 25 medical staff evaluated	− 0.475	< 0.01
Positive rate of locomotive syndrome	− 0.268	< 0.01
Hemoglobin (g/l)	0.262	< 0.01
ALB (mg/dl)	0.443	< 0.01
Total P1NP (μg/l)	− 0.285	< 0.01
TRACP-5b (mU/dl)	− 0.366	< 0.01
BMD proximal femur (g/cm^2^)		
Factor	*rβ*	*p* value
Age	− 0.437	< 0.01
Female	− 0.360	< 0.01
BMI	0.638	< 0.01
Inpatient	− 0.473	< 0.01
The number of fractured vertebrae (L-spine)	− 0.527	< 0.01
The score of locomo 25 medical staff evaluated	− 0.459	< 0.01
Positive rate of locomotive syndrome	− 0.295	< 0.01
Hemoglobin (g/l)	0.383	< 0.01
ALB (mg/dl)	0.548	< 0.01
Ca (mg/dl)	0.213	0.021
Total P1NP (μg/l)	− 0.387	< 0.01
TRACP-5b (mU/dl)	− 0.435	< 0.01

To control for any confounding variables, we performed a multiple regression analysis. In this analysis, we set the BMD of the lumbar spine or proximal femur as the dependent variable. The factors found to be significantly correlated with the BMD in the lumbar spine were the age, sex, BMI, the number of fractured vertebrae in the lumbar spine, and the medical staff-evaluated score of the locomo 25. The serum concentrations of PINP and TRACP-5b and the treatment form were not correlated with the BMD in the lumbar spine. Similarly, the factors found to be significantly correlated with the BMD in the proximal femur were also the sex, BMI, serum concentration of Alb, and the number of fractured vertebrae in the lumbar spine (Table 3). As with the lumbar spine, the serum concentrations of PINP and TRACP-5b and the treatment form were also not correlated with the BMD in the proximal femur.

**Table 3:** The results of multiple regression analysis

BMD lumbar spine (g/cm^2^)		
Factors	β	*p* value
Age	− 0.17	0.024
Female	− 0.32	< 0.01
BMI	0.29	< 0.01
The number of fractured vertebrae (L-spine)	− 0.16	0.031
The score of locomo 25 medical staff evaluated	− 0.22	< 0.01
BMD Proximal femur (g/cm^2^)		
Factors	β	*p value*
Female	− 0.29	< 0.01
BMI	0.40	< 0.01
Alb	0.24	< 0.01
The number of fractured vertebrae (L-spine)	− 0.25	< 0.01

## Discussion

In the present study, the BMI, the state of the bone density, the physical function, and the serum concentrations of Hb, Alb, and Ca were lower in the inpatient group than in the outpatient group. However, the age, the number of fractured vertebrae, the risk ratio of proximal femur fracture and major osteoporotic fracture, and the serum concentration of TRACP-5b were higher in the inpatient group than in outpatient group.

As shown in Table 1, the BMI was significantly lower and the rate of administration of typical antipsychotic drug significantly higher in the inpatient group than in the outpatient group, while the CP equivalent was not markedly different between the two groups. In addition, the physical function was lower in the inpatient group than in the outpatient group. Previous studies have found that the BMI correlated positively with the BMD [14–16], and weight gain is a well-known side effect of typical antipsychotic agents [17, 18]. However, another study reported that the degree of weight gain as a side effect of atypical antipsychotic agents differs among agents [18, 19]. We therefore expected the weight gain and BMD to be higher in the inpatient group than in the outpatient group; this was not what we actually observed, as the inpatient group had a lower BMI and BMD than the outpatient group. These results may be derived from two major differences between the inpatient and outpatient groups. First, the nutritional status differed markedly between the two groups. A sub-analysis of the present study showed a positive correlation between the CP equivalent and the BMI (*r* = 0.374, *p* < 0.01) in the inpatient group, whereas these parameters were not correlated in the outpatient group, suggesting that the inpatient group was managed with a controlled nutritional status while the outpatient group had no dietary restrictions. Second, the physical function differed markedly between the two groups. The physical function, as evaluated by the locomo 25, was significantly lower in the inpatient group than in the outpatient group, and the positive rate of locomotive syndrome was significantly higher in the inpatient group than in the outpatient group. Thus, disuse atrophy based on a low physical function may have affected the weight loss in the inpatient group.

As shown in Table 1, the BMD, YAM, and T score at the lumbar spine and proximal femur were significantly lower and the number of fractured vertebrae significantly higher in the inpatient group than in the outpatient group. In addition, the inpatient group had a higher risk of proximal femur fracture and major osteoporotic fracture than the outpatient group. Regarding osteopenia in schizophrenic patients, antipsychotic agents are thought to affect the BMD via the inhibition of hypothalamic endocrine caused by the chronic elevation of PRL [7]. However, previous studies have reported controversial findings regarding the correlation between serum concentrations of PRL and the BMD [7, 20]. BMI, by contrast, has been consistently reported to be positively correlated with the BMD in schizophrenic patients, and the serum concentration of TRACP-5b is negatively correlated with the BMD in postmenopausal women [16]. The results of the univariate regression analysis in the present study also indicated that older age, female gender, inpatient status, a higher staff-evaluated score of the locomo 25, a positive rate of locomotive syndrome in the medical staff evaluation, a higher number of fractured vertebrae, and higher serum concentration of PINP and TRACP-5b were negatively correlated with the BMD in the lumbar spine and proximal femur (Table 2). In contrast, the BMI and serum concentrations of Hb, Alb, and Ca were positively correlated with the BMD (Table 2). However, a multiple regression analysis revealed that the age, sex, BMI, the number of fractured vertebrae, and medical staff-evaluated score of the locomo 25 were significantly associated with the BMD in lumbar spine, and the sex, BMI, serum concentration of Alb, and the number of fractured vertebrae were significantly associated with the BMD in the proximal femur (Table 3). Of note, despite the BMD being significantly lower in the inpatient group than in the outpatient group, the results of the multiple regression analysis showed that the therapeutic form did not influence the BMD. Because the age and BMI were found to be a factor influencing the BMD, we considered this discrepancy to be due to the relatively high age and low BMI in the inpatient group compared with the outpatient group. The observed difference in the BMD may merely have been due to differences in the age and BMI between the inpatient and outpatient groups. In addition, the multiple regression analysis in the present study further revealed that the serum concentrations of PINP and TRACP-5b did not influence the BMD. However, these factors are indicators of the current status of bone metabolism [21, 22] and will help physicians determine appropriate anti-osteoporotic drugs.

Several limitations associated with the present study warrant mention. First, we divided the participants into two groups according to the therapeutic form. For this reason, we were unable to randomize the participants. Second, due to refusal by some participants, we were unable to obtain data on the serum concentrations of PRL from all participants. We therefore were unable to evaluate the effect of this factor on the BMD completely. Third, the small number of participants and performing our study at a single center in one region may have limited the interpretation of our results. Further studies are needed for clarifying the true factors influencing osteopenia in patients with schizophrenia.

## Conclusion

In summary, the present study revealed the actual status of osteopenia in patients with schizophrenia. The age, sex, BMI, physical function, and number of fractured vertebrae were found to be factors influencing the BMD, while the therapeutic form was not influential. Nevertheless, inpatients have a high risk of fracture based on the rate of severe osteopenia. Appropriate medications for osteopenia or rehabilitation programs may improve these risks and help at-risk patients return to the local community.
